# Deep Learning-Based Classification and Voxel-Based Visualization of Frontotemporal Dementia and Alzheimer’s Disease

**DOI:** 10.3389/fnins.2020.626154

**Published:** 2021-01-21

**Authors:** Jingjing Hu, Zhao Qing, Renyuan Liu, Xin Zhang, Pin Lv, Maoxue Wang, Yang Wang, Kelei He, Yang Gao, Bing Zhang

**Affiliations:** ^1^National Institute of Healthcare Data Science at Nanjing University, Nanjing, China; ^2^State Key Laboratory for Novel Software Technology, Nanjing University, Nanjing, China; ^3^Department of Radiology, The Affiliated Nanjing Drum Tower Hospital of Nanjing University Medical School, Nanjing, China; ^4^Medical School of Nanjing University, Nanjing, China

**Keywords:** deep learning, convolutional neural network, frontotemporal dementia, Alzheimer’s disease, MRI, visulization

## Abstract

Frontotemporal dementia (FTD) and Alzheimer’s disease (AD) have overlapping symptoms, and accurate differential diagnosis is important for targeted intervention and treatment. Previous studies suggest that the deep learning (DL) techniques have the potential to solve the differential diagnosis problem of FTD, AD and normal controls (NCs), but its performance is still unclear. In addition, existing DL-assisted diagnostic studies still rely on hypothesis-based expert-level preprocessing. On the one hand, it imposes high requirements on clinicians and data themselves; On the other hand, it hinders the backtracking of classification results to the original image data, resulting in the classification results cannot be interpreted intuitively. In the current study, a large cohort of 3D T1-weighted structural magnetic resonance imaging (MRI) volumes (*n* = 4,099) was collected from two publicly available databases, i.e., the ADNI and the NIFD. We trained a DL-based network directly based on raw T1 images to classify FTD, AD and corresponding NCs. And we evaluated the convergence speed, differential diagnosis ability, robustness and generalizability under nine scenarios. The proposed network yielded an accuracy of 91.83% based on the most common T1-weighted sequence [magnetization-prepared rapid acquisition with gradient echo (MPRAGE)]. The knowledge learned by the DL network through multiple classification tasks can also be used to solve subproblems, and the knowledge is generalizable and not limited to a specified dataset. Furthermore, we applied a gradient visualization algorithm based on guided backpropagation to calculate the contribution graph, which tells us intuitively why the DL-based networks make each decision. The regions making valuable contributions to FTD were more widespread in the right frontal white matter regions, while the left temporal, bilateral inferior frontal and parahippocampal regions were contributors to the classification of AD. Our results demonstrated that DL-based networks have the ability to solve the enigma of differential diagnosis of diseases without any hypothesis-based preprocessing. Moreover, they may mine the potential patterns that may be different from human clinicians, which may provide new insight into the understanding of FTD and AD.

## Introduction

Although the separation between the dementia group and the NC group was clear, it does not mean that an individual admitted can be accurately diagnosed. In clinical practice, after noticing dementia symptoms that cannot be explained by age factor, doctors must determine which specific dementia the patient belongs to, so as to provide targeted treatment and patient care. With the increasing incidence of dementia (Zissimopoulos et al., 2018), precise identification of FTD and AD, which are the two most common types of dementia in the younger-elderly population (Bang et al., 2015; Association, 2019), is of vital clinical significance in the diagnosis of dementias. Nevertheless, the clinicopathological correlation between FTD patients is low (Ikeda et al., 2019), and the behavioral, psychological, and medical imaging manifestations of FTD and AD patients highly overlap (Pawlowski et al., 2019). These bring great challenges to the differential diagnosis of FTD and AD. Researchers have tried to solve the above problems from various perspectives of protein (Jang et al., 2018), gene (Luukkainen et al., 2019), behavior, imaging (Tosun et al., 2016; Schiller et al., 2019), etc. Among them, the atrophy of specific brain regions shown by structural MRI is an important part of the diagnostic criteria for FTD and AD (McKhann et al., 2011; Rascovsky et al., 2011), and magnetic resonance scanning has become a standard procedure in the clinical workflow.

**FIGURE 1:**
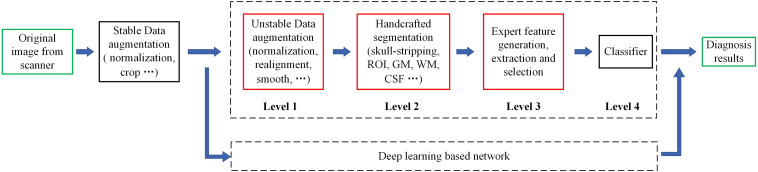
Motion of the workflow of the computer-aided diagnosis system. The green box represents the input and output data of the system, while the red box indicates that the step is fragile and requires human supervision or design. Unstable data augmentation refers to data preprocessing operations that rely heavily on specific software, specific hyperparameter settings, and even specific versions. Depending on studies, some normalization algorithms are stable and some are unstable. Similarly, some studies align volumes to MNI spaces, while others align volumes to custom templates. Most studies remove the skulls before extracting features, either by masking them or manually sketching them.

However, MRI-based diagnosis mainly relies on the professional knowledge and clinical experience of doctors, leading to unsatisfactory diagnostic accuracy, especially in small cities and small community medical centers. Machine learning (ML) has made amazing achievements in many scientific fields, especially in computer vision, natural language processing and advertising recommendation fields, which have attracted many researchers to apply it recently to medical problems. By

reviewing the research on ML in FTD and AD (Klöppel et al., 2008; Bron et al., 2017; Bouts et al., 2018; Kim et al., 2019), we can lightly find that the existing ML-aided FTD and AD differential diagnosis algorithms rely on rigorous and manual data preprocessing, feature extraction and feature selection, which are skillfully designed by experts (Figure 1). This reliance not only makes it difficult to reproduce the experimental results but also hinders the integration of the model into the actual clinical diagnosis workflow, which further leads to substantial reduction in clinical significance.

As an end-to-end network algorithm, DL no longer relies on feature engineering, which lowers the barrier to entry and promotes the sharing of cross-domain knowledge. Moreover, the designs of deep learning network in terms of the depth, width and interlayer connection enable it to explore the potential characteristics of data as much as possible. DL methods have recently shown promising results in detecting cartilage damage (Liu et al., 2018), predicting mild cognitive impairment (MCI) prognosis (Basaia et al., 2019), and identifying AD patients via conventional MRI scans (Liu et al., 2020). All these findings suggest that the differential diagnosis of FTD and AD can be solved by feeding a DL network with raw 3D MRI data without any neuroanatomist level preprocessing, which, to our knowledge, has not yet been done.

Furthermore, there is still a wide gap between the application of DL in scientific research and its application in clinical practice. One reason is that although the input data are not expert-level preprocessed, they are still carefully selected (Burgos and Colliot, 2020), which results in a small sample size and poor generalizability of the model. In addition, existing DL-assisted diagnostic studies still rely on hypothesis-based expert-level preprocessing, which, on the one hand, imposes high requirements on clinicians and data themselves, and on the other hand hinders the backtracking of classification results to the original image data, resulting in the classification results cannot be interpreted intuitively.

**FIGURE 2:**
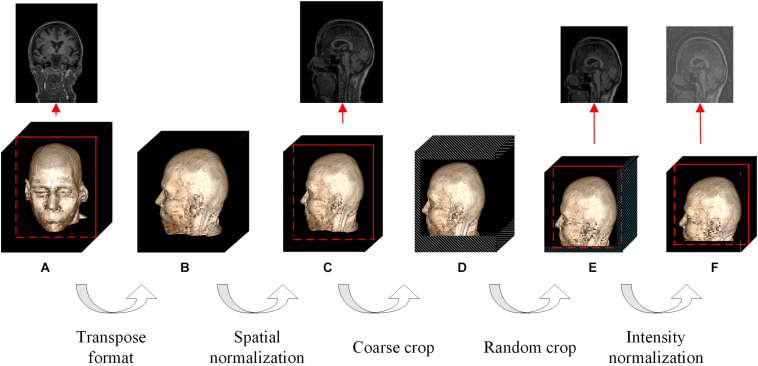
Flowchart of the whole data augmentation module. The stereo block at the bottom represents a 3D T1-weighted structural MRI volume. The red line in the block indicates that a slice is taken from the corresponding position and displayed on the top. The white stripe area in the block indicates that these positions will be clipped directly, and the blue stripe area indicates that the offset starting position is selected randomly at these positions. **(A)** The original image in the datasets can be in any format and any size. **(B)** The multi-center 3D T1-weighted structural MRI volumes are all converted to DHW format. **(C)** The size of spatial normalized image is 240 × 256 × 160 pixels. **(D)** The size of coarse cropped image is 232 × 200 × 160 pixels. **(E)** The size of cropped image is 224 × 192 × 160 pixels. **(F)** The image obtained by intensity normalization is feed to the network for training.

**FIGURE 3:**
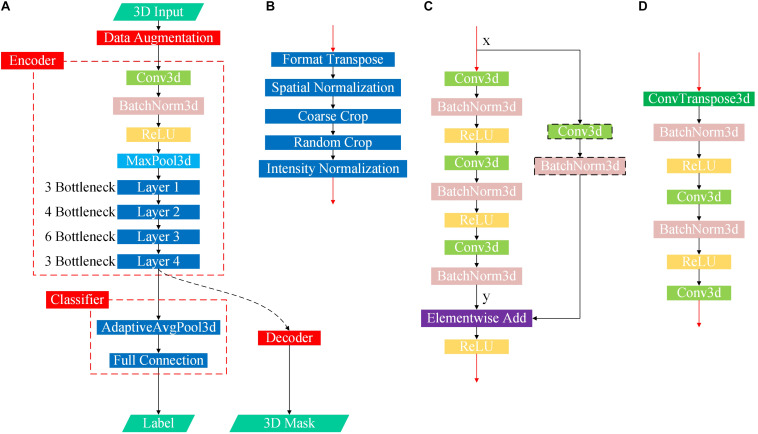
Flowchart of the proposed DL-based framework. **(A)** First, we learned universal feature representations by training a segmentation network composed of data augmentation, encoder and decoder modules. Second, we transferred the backbone network part from the segmentation task as the feature extraction part and then learned and trained the classifier. **(B)** Flowchart of the data augmentation module. **(C)** Flowchart of the bottleneck submodule. Layers 1–4 in the encoder are cascaded by several bottleneck submodules, and the first bottleneck of each layer has a downsampling module before the elementwise addition operator to ensure that the two matrix dimensions to be added are consistent, as shown in the black dotted box, while the other bottleneck submodules directly add x and y. **(D)** Flowchart of the decoder.

We solve the above puzzles from two aspects: training DL-based networks without any hypothesis-based preprocessing (Figure 2) and testing their differential diagnosis ability for FTD and AD; visualizing the contribution graph of each sample and explaining the basis of network decision-making. In the first step, a large number of samples were collected according to a loose constraint, among which 1,314 AD patients and 938 NCs were obtained from the ADNI database, while 1,250 FTD patients and 597 NCs were obtained from the NIFD database. Second, we initialized the networks with a pretrained model (Chen et al., 2019) and trained the classifier (Figure 3) on the loose dataset. Third, we tested the classification capability in the independent datasets and calculated the corresponding contribution graph for each sample. In the last step, affine matrixes mapping the original 3D MRI volumes into the standard Montreal Neurological Institute (MNI) space were used to visualize the overall contribution graph of each category. To facilitate the community to reproduce our experimental results based on the same data and methodology or apply our network to other applications, we have released our source code, relative pre-trained models and logs^1^.

## Materials and Methods

### Data Collection

In contrast to previous studies, the 3D T1-weighted structural MRI data used in this study were collected from two open accessible databases with looser inclusion criteria, which is conducive to driving DL networks to obtain better classification performance, guaranteeing the diversity of data and the generalization ability of the model. Under the guidance of the same inclusion criteria, we collected FTD patient data from the NIFD database, AD patient data from the ADNI database, and normal control group data from both databases (abbreviated FTD_NC and AD_NC, respectively).

Based on the most common T1-weighted sequence (MPRAGE), the sample sizes of AD, AD_NC, FTD, and FTD_NC were 422, 469, 552, and 354, respectively. It should be noted that even if the AD patients in the ADNI database and the FTD patients in the NIFD can be distinguished, it is difficult to explain whether the classification ability of DL is based on the diseases themselves or on the different databases.

FTLDNI was funded through the National Institute of Aging, and started in 2010. The primary goals of FTLDNI were to identify neuroimaging modalities and methods of analysis for tracking FTD and to assess the value of imaging vs. other biomarkers in diagnostic roles. The Principal Investigator of NIFD was Dr. Howard Rosen, MD at the University of California, San Francisco. The data are the result of collaborative efforts at three sites in North America. For up-to-date information on participation and protocol, please visit http://memory.ucsf.edu/research/studies/nifd.

The ADNI was launched in 2003 as a public-private partnership, led by Principal Investigator Michael W. Weiner, MD. The primary goal of ADNI has been to test whether serial magnetic MRI, positron emission tomography (PET), other biological markers, and clinical and neuropsychological assessment can be combined to measure the progression of MCI and early AD. For up-to-date information, see www.adni-info.org.

The collected MRI volumes must meet all the following criteria: the scanning plane is the sagittal plane, the field strength is equal to 3 tesla, the slice thickness is between 0.8 and 1.5 mm, and the number of slices is between 150 and 250. It should be noted that we do not limit the patient’s age, gender, weight and other personal information, nor restrict the manufacturer, coil and other scanning parameters. We also do not perform any selection or quality control of the volumes, such as having medical experts check the image quality.

Although we filtered the databases using loose inclusion criteria, the collected data were still very diverse: volumes were scanned by scanners from 3 manufacturers (Philips Medical Systems, SIEMENS and GE Medical Systems) with different slice thickness (1.0 and 1.2 mm), resulting in heterogeneous dimensions. Considering that we did not perform any manual preprocessing (such as non-brain tissue removal, substantial tissue segmentation, standard MNI space transformation, non-uniformity correction, quality control, etc.), it is extremely challenging to classify such a complex dataset.

Based on these two databases, we firstly designed 4 experimental scenarios to evaluate the convergence speed, differential diagnosis ability, generalizability and robustness of our network, as shown in Table 1. Considering that the same subject may be scanned multiple times at multiple time points, once the test data participates in the training process in any form, it will cause data leakage and result in unreasonable model evaluation. Therefore, the loose datasets were randomly divided into training datasets and testing datasets at the subject level according to a ratio of 4:1. The test results based on these independent data can objectively quantify the generalization ability of the model.

**TABLE 1:** Experimental scenarios.

Train objectives	Test objectives	Scenario	Test accuracy (%)	Details
FTD vs. FTD_NC	FTD vs. FTD_NC	1	93.45	Figure 5
AD vs. AD_NC	AD vs. AD_NC	2	89.86	Figure 5
FTD vs. AD vs. NC	FTD vs. AD vs. NC	3	91.83	Figure 5
	FTD vs. AD	4	93.05	Figure 8C

*The train datasets of scenario 3 and scenario 4 overlap to further evaluate the generalizability of the network.*

### Data Augmentation

The contradiction lies in that the diversity of the data is helpful for improving the accuracy and robustness of the network, while the inconsistency of the data makes it more difficult for the network to fit the pattern and prevents network convergence. To solve this dilemma, we first fed all the images into the data augmentation module (Figure 2), where the image spatial scale and pixel intensity were normalized, and then the enhanced data were sent to the baseline network.

For the convenience of the following description, D denotes the depth from the anterior to the posterior head, H denotes the height, and W denotes the width. In the first step, the multicenter 3D T1-weighted structural MRI volumes were all converted to DHW format, followed by resampling all the images to a fixed size of 240 × 256 × 160 pixels in DHW format to complete the spatial normalization step. To avoid over interpolation, zoom was going to fill in for the missing values with spline interpolation algorithm of order 0.

In the field of DL, random cropping of images can further expand the sample space, weaken data noise, and improve the robustness of the network. Nevertheless, common random cropping and random center cropping tend to miss important brain structures, which is attributed to the fact that cranial MRI volumes have little redundancy in the left, right, anterior and posterior sides, while the upper and bottom (cervical) sides usually have large redundancy. In this work, the resampled images were coarsely cropped by 8, 16, and 40 pixels at the anterior, upper, and bottom boundaries, respectively, and then randomly offset by 0–8 pixels in the horizontal and vertical directions to obtain a fixed size of 224 × 192 × 160 pixels.

In addition, collection from different devices, different protocols, and different scanning parameters resulted in our multicenter data not meeting the assumption of a statistically identical distribution in terms of the numerical intensity. Thus, we normalized the intensity value *v*_*i,j,k*_ based on the mean *v_m* and the standard deviation *v*_*std*_ of nonzero region in the individual volume at the end of our data augmentation module as:

(1) $$\begin{array}{c} v_{i,j,k}^{\prime }=\frac{v_{i,j,k}-v_{m}}{v_{std}} \end{array}$$

### Network Architecture and Transfer Learning

One of the research purposes of this paper is to verify whether DL is sufficiently competent for the FTD and AD classification tasks without manual intervention by medical experts. Therefore, we chose a common baseline network (Chen et al., 2019) that has been proven to be effective in multiple tasks, and concentrated on the classification problem itself and the visual interpretation of the network.

The data augmentation methods used in this paper is stable and require few knowledges of clinical medicine. All operations of the whole data augmentation module were written in Python and released in the source code. Users can download the raw data from the public database (ADNI, NIFD or other customized database) and feed it to the network directly without additional manual modification.

In practice, we do not need to train an entire network from scratch because initializing the network with a pretrained model that has been trained with relatively large datasets can significantly accelerate the training convergence, reduce overfitting, and improve the accuracy to some extent (Tajbakhsh et al., 2016).

The original study (Chen et al., 2019) selects data from segmentation datasets to train the network, but our task is solving the classification problem. Therefore, we modified the baseline network by the following four points: (1) replacing the data augmentation module; (2) transferring the encoder; (3) discarding the decoder; and (4) adding a classifier (Figure 3).

The detailed design and parameters of the data augmentation module have been discussed in section “Data Augmentation.” When the batch size (denoted by N) for one training was set to 12, the output data dimension of the module was 12 × 1 × 224 × 192 × 160 in NCDHW format (where C represents the number of channels). The prototype of the reused encoder was actually 3D-ResNet50, whose detailed parameters and source code have been released, and the output data dimension was 12 × 2,048 × 28 × 24 × 20. The AdaptiveAvgPool3d operator in the classifier pooled the DHW data to scalar. Considering that the problem discussed in this paper is the multiclassification of AD, FTD and NC, the output data dimension of the designed classifier was 12 × 3, indicating the probability of the 12 samples selected in the current training batch belonging to the three categories.

All the network models were trained on a DGX-1 hardware platform, and the software frameworks were PyTorch 1.2.0, Python 3.6.9, and CUDA 10.0. The whole network optimizer was the stochastic gradient descent algorithm, for which the momentum factor was 0.9 and the weight delay factor was 0.001. The loss function of the whole network was:

(2) $$\begin{array}{c} \text{loss}\left(I_{i},t\right)=-\log\left(\frac{exp\left(out_{i}^{t}\right)}{\sum_{t=0}^{K-1}exp\left(out_{i}^{t}\right)}\right) \end{array}$$

where I_*i*_ denoted the input image, K represented the total number of categories, and $out_{i}^{t}$ was the score of I_*i*_ belonging to label t.

The ground truth of the sample was marked by the public database, and the diagnostic criteria were specified by ADNI and NIFD. For example, the inclusion criteria of AD in ADNI are^2^ : MMSE (Mini-mental State Examination) scores between 20 and 26 (inclusive), CDR (Clinical Dementia Rating) of 0.5 or 1.0, and meets NINCDS (National Institute of Neurological and Communicative Disorders and Stroke)/ADRDA (Alzheimer’s Disease and Related Disorders Association) criteria for probable AD. The predicted label of the sample was finally calculated by the classifier in the model. For a particular sample, the model output the probability that the image belongs to each category, and the category corresponding to the maximum probability was the final predicted value.

The initial learning rate of the whole network was 0.001, and the learning rate scheduler was a cosine annealing algorithm, so the learning rate of each training batch was:

(3) $$\begin{array}{c} \eta_{t}=\eta_{min}+\frac{1}{2}\left(\eta_{max}-\eta_{min}\right)\left(1+cos\left(\frac{T_{cur}}{T_{max}}\pi \right)\right) \end{array}$$

where η_*max*_was set to the initial learning rate 0.001, and η_*min*_ was 0. *T*_*cur*_ was the number of epochs since the last restart, and *T*_*max*_ was 5.

### Contribution Calculation

Even though DL networks can be well qualified for the classification task, it is difficult for people to understand how network makes the right decision. With the deepening of the network and the cascading of various operators, the high-level feature map becomes increasingly abstract. To understand the decision strategy of the DL network, a magic black box, and to verify the rationality and physiological mechanism of the classification network, this paper applied a gradient visualization algorithm based on guided backpropagation (Springenberg et al., 2014) to calculate the contribution graph.

The core theory of this algorithm as follows: given an input image I_*i*_ and a target label t, the contribution graph $C_{i}^{t}$is obtained by guided backpropagating the gradient from the top layer to the bottom layer. The difference between the guided backpropagation in this algorithm and ordinary backpropagation lies in the gradient of the activation function $R_{i}^{l}$being replaced by the contribution $C_{i}^{l}$:

(4) $$\begin{array}{c} f_{i}^{l+1}=ReLU\left(f_{i}^{l}\right)=max\left(f_{i}^{l},0\right) \end{array}$$

(5) $$\begin{array}{c} R_{i}^{l}=\left(f_{i}^{l}>0\right)\cdot R_{i}^{l+1},whereR_{i}^{l+1}=\frac{\partial f^{out}}{\partial f_{i}^{l+1}} \end{array}$$

(6) $$\begin{array}{c} C_{i}^{l}=\left(f_{i}^{l}>0\right)\cdot \left(R_{i}^{l+1}>0\right)\cdot R_{i}^{l+1} \end{array}$$

where l denotes the l-th layer, i indicates the i-th sample and f represents the feature map.

As shown in the equations, $C_{i}^{l}$ adds a constraint to $R_{i}^{l}$, which inhibits the backpropagation of the negative gradient item and prevents the participation of neurons that reduce the activation value.

### Model Visualization

In conclusion, given an input image I_*i*_ and a target label t, we will get a one-to-one correspondence contribution graph $C_{i}^{t}$:

(7) $$\begin{array}{c} C_{i}^{t}=G\left(I_{i},t\right) \end{array}$$

**FIGURE 4:**
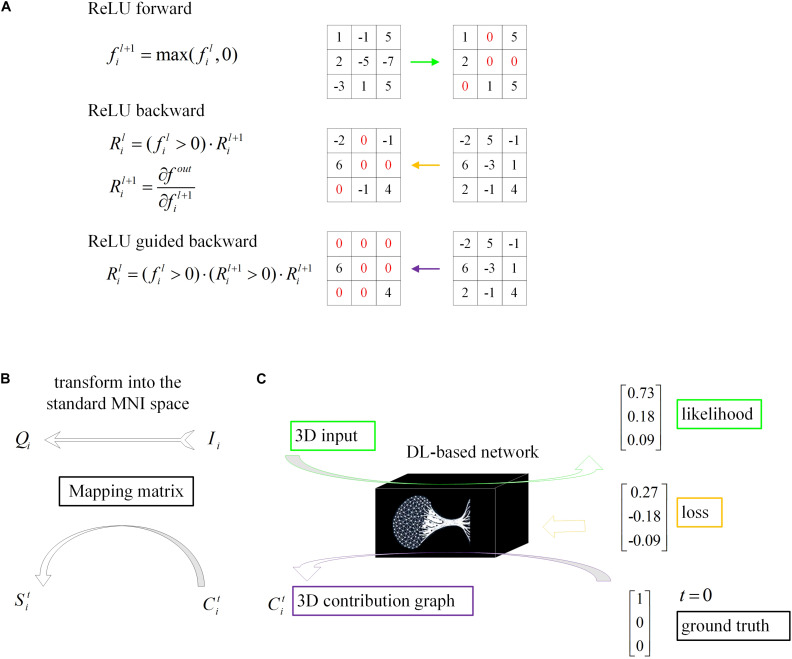
Schematic of model visualization. **(A)** Difference methods of propagation forward and backward through a ReLU nonlinearity layer. **(B)** The 3D contribution graph was transformed into the standard MNI space according to the corresponding raw structural MRI image. **(C)** The difference between the guided backpropagation (information flow in purple) in this algorithm and ordinary backpropagation (information flow in yellow).

The dimensions of the samples in our loose dataset are different, resulting in different dimensions of the contribution graph (Figure 4). Therefore, a reliable transformation method is needed to integrate all the contribution graphs of each category to observe and explain its statistical laws. Every image I_*i*_ was first transformed into the standard MNI space using Statistical Parametric Mapping (Penny et al., 2011) to obtain the mapping matrix M_*i*_, which was then used to map contribution graph $C_{i}^{t}$ to $S_{i}^{t}$.

The unique contribution graph of each classification label *S*^*t*^ is calculated by:

(8) $$\begin{array}{c} S^{t}=\frac{1}{N}\sum_{i=0}^{N-1}S_{i}^{t} \end{array}$$

where N denotes the total number of samples labeled t. Results were represented by AFNI (Cox, 1996).

The final visualized maps showed *Z*^*t*^ values which defined as the raw average contribution score *S*^*t*^minus the mean of the whole brain and then divided by the standard deviation. This Z transformation is to enhance the contrast given the average contribution score is basically uniformed across the whole brain. Moreover, to investigate how the scores were difference between AD and FTD, we performed a two-sample *t-*test, and significance threshold was set to 0.0001.

## Results

### Convergence Speed

**FIGURE 5:**
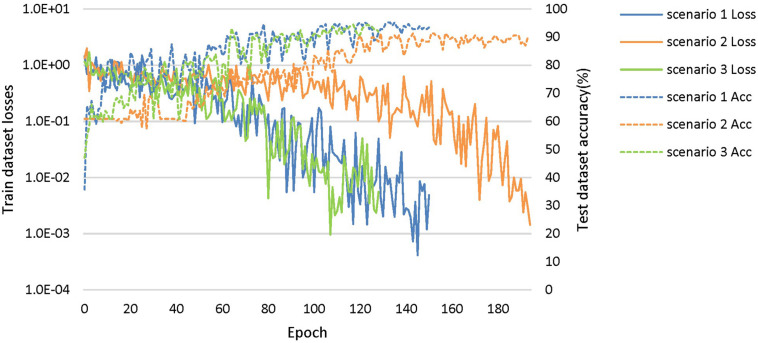
The training process of the proposed network under scenarios 1, 2, and 3. The losses curves in train datasets are represented by solid lines in the figure, while the accuracy curves in test datasets are represented by dashed lines.

The loss curves of the training process (Figure 5) reflect the convergence speed of the corresponding scenario. Referring to the dataset composition of each scenario, the curves help us understand the learning law of training the DL network. In scenarios 1 and 2, the training sample sizes were 725 and 712, respectively (the training sample size accounted for approximately 80% of the total sample size), which were roughly equivalent. Comparing the solid blue line of scenario 1 and the solid orange line in scenario 2, it can be seen that the loss value of AD during training still fluctuated after approximately 100 epochs, while the loss value of FTD was basically stable below 0.001. In scenario 3, the training sample size was 1,437, which was the sum of scenarios 1 and 2. The DL network tended to converge after training with about 1,80,000 images (130 epochs) in scenario 3 and 1,00,000 images (150 epochs) in scenario 1.

### Diagnostic Accuracy

The accuracy curves of each scenario in the independent test datasets (Figure 5) reflect the classification performance of the corresponding scenario, which is conducive to understanding the capacity of the proposed network. The accuracy of scenario 3 (91.83%) was lower than the weighted average accuracy of scenarios 1 and 2 (93.45 and 89.86%, respectively). Note that the training samples from scenario 3 came from two open datasets, and the NC images were also a collection of the corresponding NC images from the two open datasets, making the multiclassification task more difficult than recognizing the disease itself.

### Results on Visualization

The voxel-based contribution map helps clinical radiologist understand the abstract DL network and more confidently evaluate the justifiability and reference value of objective decision making given by the DL network.

**FIGURE 6:**
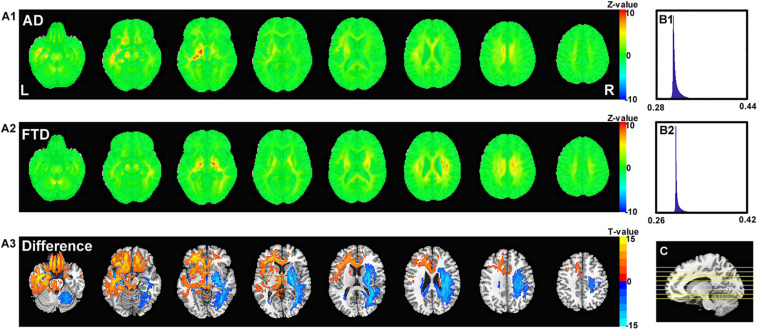
The calculated unique contribution graphs of each disease. **(A1)** the mean contribution in each voxel of all AD subjects. **(A2)** the mean contribution in each voxel of all FTD subjects. Both **(A1,A2)** showed *Z*-value. (**A3**) the AD-FTD difference of the contribution maps, here showing T-value of two sample *t*-test and regions have *p* < 0.0001. (**B1)** the histography of the mean contribution of AD group. (**B2)** the histography of the mean contribution of FTD group. **(C)** the location of the axial slices shown in **(A1–A3)**.

Figure 6 shows the visualization results of the classification. First, we found that the contribution scores were quite uniformed across the whole brain, and the histography showed a very narrow spike around 0.3 for both AD and FTD (Figures 6B1,B2). However, there were still some specific regions showing higher contribution compared to other regions. For the AD group, the high-contribution regions were focused on the corpus callosum, cingulate cortex, subcortical regions, left hippocampus and white matter around it. For the FTD group, the high-contribution regions located in subcortical regions, the corpus callosum and the white matter under the right frontal lobe (Figures 6A1,A2). Two sample *t*-test showed that widespread regions including inferior left temporal lobe, bilateral inferior frontal lobe, hippocampus, thalamus and medial frontal cortex may contribute more to classifying of AD subjects compare to FTD. In contrast, the widespread white matter regions in the right hemisphere contributed in FTD significantly more than AD (Figure 6A3).

### Generalizability

For retrospective studies, the multicenter dataset accessed by researchers are often diverse due to historical factors such as device replacement, technology development and operation inconsistency. For example, under the premise of meeting the inclusion criteria described in section “Data Collection,” the ADNI images still scanned from more than 30 kinds of protocols [MPRAGE, spoiled gradient-recalled echo (SPGR), fast SPGR (FSPGR), etc.]. To further assess the generalizability of DL networks in extremely harsh environments, we removed the restriction of scanning from MPRAGE sequence and collected some looser datasets (Table 2).

**TABLE 2:** The demographic and scan parameters of the looser datasets.

Class	Database	Num (train/test)	Age (mean ± std)	Male/female	Flip angel (degrees)	Slice thickness (mm)
AD	ADNI	334/88	75.5 ± 7.79	230/192	9.00 ± 0	1.20 ± 0
FTD	NIFD	440/112	65.1 ± 7.48	332/220	9.00 ± 0	1.20 ± 0
NC	ADNI	381/88	75.3 ± 6.19	224/245	9.00 ± 0	1.20 ± 0
	NIFD	282/72	64.9 ± 7.85	151/203	9.00 ± 0	1.20 ± 0
AD^*l*^	ADNI	1,051/263	75.4 ± 8.02	729/585	9.16 ± 1.20	1.18 ± 0.05
FTD^*l*^	NIFD	1,001/249	64.9 ± 7.63	744/506	8.93 ± 0.26	1.20 ± 0
NC^*l*^	ADNI	749/189	75.7 ± 6.64	458/480	9.08 ± 1.08	1.16 ± 0.08
	NIFD	475/122	64.4 ± 8.17	240/357	9.00 ± 0	1.20 ± 0

*The superscript “l” indicates that we removed the restriction of scanning from MPRAGE sequence and distinguish from scenarios 1–4.*

**FIGURE 7:**
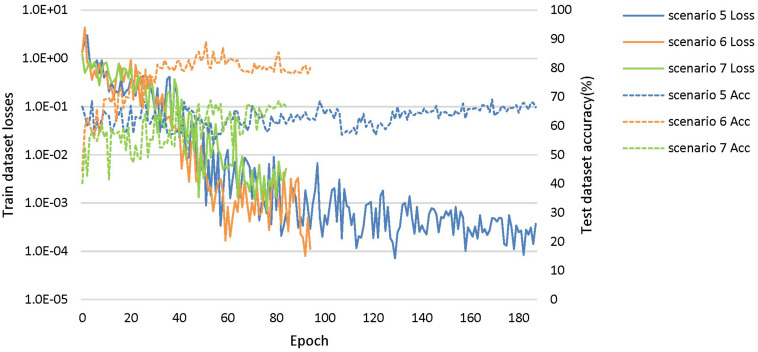
The training process of the proposed network under scenarios 5, 6, and 7. The losses curves in train datasets are represented by solid lines in the figure, while the accuracy curves in test datasets are represented by dashed lines.

Based on these looser databases, we further designed 5 experimental scenarios (Table 3) and compared them with performance under scenarios 1–4 (Figure 7).

**TABLE 3:** Experimental scenarios in looser datasets.

Scenario	Train objectives		Test objectives		Test accuracy (%)
	Data component	Data amount	Data component	Data amount	
1	FTD, FTD_NC	440, 282	FTD, FTD_NC	112, 72	93.45
2	AD, AD_NC	334, 381	AD, AD_NC	88, 88	89.86
3	FTD, AD, NC	440, 334, 282+382	FTD, AD, NC	112, 88, 72+88	91.83
4	FTD, AD, NC	440, 334, 282+382	FTD, AD	112, 88	93.05
5	FTD^*l*^, FTD_NC^*l*^	1,001, 475	FTD^*l*^, FTD_NC^*l*^	249, 122	68.02
6	AD^*l*^, AD_NC^*l*^	1,051, 749	AD^*l*^, AD_NC^*l*^	263, 189	77.18
7	FTD^*l*^, AD^*l*^, NC^*l*^	1,001, 1,051, 475+749	FTD^*l*^, AD^*l*^, NC^*l*^	249, 263, 122+189	66.79
8	FTD^*l*^, AD^*l*^, NC^*l*^	1,001, 1,051, 475+749	FTD^*l*^, AD^*l*^	249, 263	81.25
9	FTD^*l*^, AD^*l*^, NC^*l*^	1,001, 1,051, 475+749	FTD, AD	112, 88	98.61

*Among them, the train dataset of scenario 7 was the same as that of scenarios 8 and 9. This design was intended to evaluate the generalizability of the network. The test datasets of scenario 9 and scenario 4 overlapped to further measure the robustness of the network. See Table 2 for the sample size of each data component. For example, in scenario 6 of Table 3, the training sample size and the test sample size corresponding to AD^*l*^ are shown in the first row in the main body of Table 2, i.e., 1,051 and 253, respectively. The superscript “l” indicates that we removed the restriction of scanning from MPRAGE sequence and distinguish from scenarios 1–4.*

The dataset of scenario 5 consisted of 1,250 FTD images and 597 NC images from the same age group. The number of positive samples was approximately two times the number of negative samples. This kind of data imbalance problem increases the training difficulty of the DL network and makes the classification performance poor. The samples in scenario 6 came from 3 manufacturers and 30 different scan protocols, and the slice thickness and dimensions between images were also greatly different, which brought enormous challenges to the classification task. Whereas, experiments showed that the DL network can still achieve 77.18% accuracy without any medical expert level preprocessing. With exactly the same network structure, training strategy and initialization parameters, the accuracy of DL network in scenarios 5, 6, and 7 was 12.7∼25.43% lower than that of the control group (scenario 1, 2, and 3 respectively). In scenario 7, the training sample size was 3,279, and the DL network tended to converge after training with about 2,60,000 images (80 epochs), compared with about 1,80,000 images (130 epochs) in scenario 3.

The knowledge learned by the network through multiclassification tasks should also be able to solve subproblems, which should be generalizable rather than limited to a specified dataset. We further fixed the encoders learned in scenarios 3 and 7, and replaced the classifier with the binary classifier of the subproblem (scenarios 4, 8, and 9) to evaluate the generalizability of the previously learned patterns.

**FIGURE 8:**
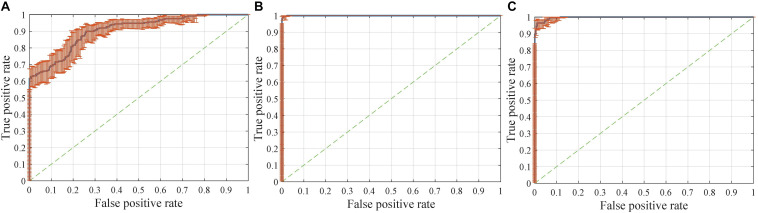
Receiver operating characteristic (ROC) curves with pointwise confidence bounds. The sensitivity (true positive rate) here reflects the percentage of FTD patients who were correctly classified having FTD against a background group of patients with AD, and the specificity (false positive rate) reflects the percentage of AD patients correctly classified as having AD. **(A)** ROC curve for FTD^*l*^ vs. AD^*l*^ in scenario 8. **(B)** ROC curve for FTD vs. AD in scenario 9. **(C)** ROC curve for FTD vs. AD in scenario 4.

Interestingly, compared with identifying FTD patients from NC, the network has a stronger ability to differentiate FTD from AD (Figure 8A). This finding implies two points: (1) the difference between FTD and AD is more obvious and easier to learn than that between FTD and NC; (2) the task of clinical radiologist is more arduous when patients do not realize they have the diseases.

The dataset for the Figure 8B task was a subset of the dataset for the Figure 8A task, with better classification performance (98.61%). The test dataset for the Figure 8B task and Figure 8C task were identical, but the encoders were different. As shown in the figures, the knowledge acquired from scenario 7 with a larger sample size and more diverse data was more universal and had the potential to be applied to new tasks.

**FIGURE 9:**
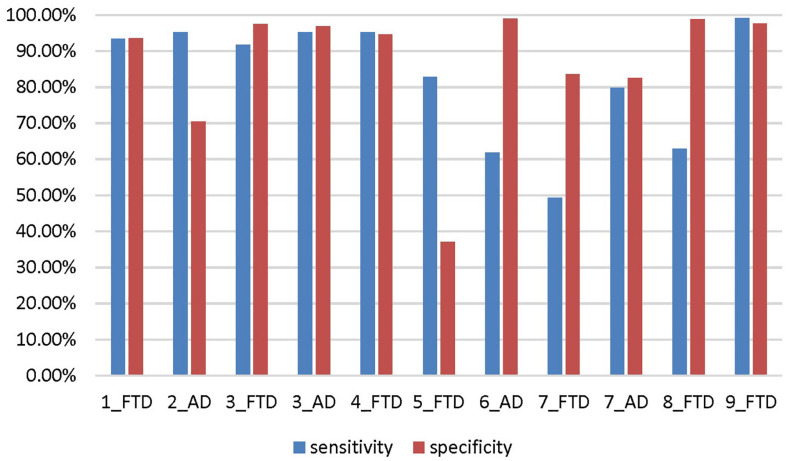
The sensitivity and specificity in addition to the accuracy for the different scenarios. The number before the underline in the abscissa label indicates the scenario, and the category after the underline indicates which disease is the positive sample. Both scenario 3 and scenario 7 are multi-classification problems, so we calculate them from the perspective of FTD as positive sample and AD as positive sample, respectively.

The accuracy, sensitivity and specificity of scenario 5 were all lower than those of scenario 1 (Figure 9), and it was especially easy to misjudge the NC samples as FTD. Similarly, the performance of scenario 7 was inferior to that of scenario 3. In scenario 6, about one third of AD patients were classified as normal controls, resulting in lower sensitivity and accuracy than in scenario 2. Although the specificity of both scenarios 8 and 9 were high, the negative samples of these two scenarios were AD rather than NC. The specificity of FTD was equivalent to the sensitivity of AD, and the classification efficiency must be considered comprehensively.

## Discussion

In summary, the foundational aim of this study is to investigate and visualize the diagnostic value of the DL-based networks in differentiating between patients with FTD, patients with AD and NCs, on an individual patient basis. The classification results showed that the proposed approach achieved promising performance without any manual intervention by medical experts. The pattern knowledge learned by the DL network is generalizable, and could be transferred to other datasets and tasks. The voxel-based contribution map results in turn showed that the networks mine the potential patterns that may be different from human clinicians.

### Inherent Drawback of Studies Based on Traditional ML

Traditional ML techniques, including logistic regression, support vector machine (SVM), principal component analysis (PCA), linear discriminant analysis (LDA) and random forests, have been used in the field of brain disorders for more than 10 years (Table 4). Their common shortcoming is that each task to be solved requires a specific, sophisticated, and time-consuming manual design, which requires researchers to explore endless problems strenuously.

**TABLE 4:** Summary of the previous machine learning based studies.

Study	Database	Num	Level 1	Level2	Level 3	Level 4	Task	Accuracy (%)
Meyer et al. (2017)	German consortium for FTD (Otto et al., 2011), local	<100	MNI	Mask, ROI, GM	VBM	SVM	bvFTD, NC	84.6
Bron et al. (2017)	local	<50	Alignment	Mask, GM, WM	VBM	SVM	AD, FTD, NC	76.2
Feis et al. (2018)	local	<100	Field correction, MNI,	Mask, ROI, GM, WM	VBM, ICA	Elastic regression net	early-FTD, NC	0.68 (AUC)
Kim et al. (2019)	local	<150	Smooth	Mask, ROI, WM	Cortical thickness	PCA, LDA	CN, FTD+AD	86.1
Oh et al. (2019)	ADNI	<200	MNI	None	CNN-based model		AD, NC	86.6
Korolev et al. (2017)	ADNI	<100	Alignment	Mask	VoxCNN, VoxResNet		AD, NC	79.1; 80.1
Liu et al. (2020)	ADNI	<100	Alignment	Mask	Multi-task DCNN		AD, NC	88.9
Basheera and Ram (2020)	ADNI	<100	Smooth	Mask, GM	Enhanced ICA+2D CNN		AD, MCI, NC	86.7
Basaia et al. (2019)	ADNI	<300	MNI	GM	CNN-based model		AD, NC	99.2
Ours	ADNI, NIFD	>400	None	None	CNN-based model		(FTD, AD, NC); (FTD, AD)	91.8; 93.1
Ours	ADNI, NIFD	>1,200	None	None	CNN-based model		(FTD^*l*^, AD^*l*^, NC^*l*^); (FTD^*l*^, AD^*l*^)	66.8; 81.3

*Num, maximum number of patients with one disease; mask, skull-skipping; bvFTD, behavioral variant FTD; early-FTD, presymptomatic FTD; GM, gray matter; WM, white matter; ICA, independent component analysis. The superscript “l” indicates that we removed the restriction of scanning from MPRAGE sequence.*

The traditional ML algorithm is mainly used at Level 4 (Figure 1), playing an important role in dimensionality reduction, voting or classification of the previously extracted feature map. This kind of auxiliary algorithm merely scratches the surface and cannot eliminate its dependence on professional knowledge and human intervention. Therefore, there is an urgent need for an algorithm that can automatically mine features from massive data, and this property is the advantage of DL.

### Shortcoming of Previous Studies Based on DL

Many studies have applied DL networks to the fields of brain region of interest (ROI) segmentation, dementia diagnosis, and disease prediction and have made considerable progress. Recent work has demonstrated that residual and plain convolutional neural networks (CNNs) (Korolev et al., 2017) based on the ADNI dataset achieve similar performance in AD classification tasks. Another study proposed a multi-model DL framework based on CNN for joint automatic hippocampal segmentation and AD classification (Liu et al., 2020). Some studies sliced natural 3D volumes into multiple slides of 2D images (Basheera and Ram, 2020) and achieved acceptable performance. Compared with this paper (Table 4), these existing studies have the following shortcomings: (1) the consistent sample size is limited, which does not conform to the actual clinical environment; (2) the manual intervention in Level 1 and Level 2 is not completely abandoned; and (3) the studies mainly concentrated on patients with AD, patients with MCI, and NC, and these methods have not been used in FTD-related research.

### What Are the Features Used by the DL Network?

Interpretability is one of the most common limitations of DL studies on medical images. In the current study, we utilized a gradient visualization algorithm based on guided backpropagation and showed the contribution weight of the classification in both the AD and FTD groups. Generally, both AD and FTD showed a uniformly distribution across the whole brain, indicating that DL may not only focused on the features from some specific regions. However, there were still high contribution region in both groups. The AD and FTD images shared some high-contribution regions, such as the subcortical regions, corpus callosum and cingulate cortex. We can speculate that the features in these regions were used to classify them from NC images given the weight were calculated from the 3-group classification, which is consistent with previous studies indicating that both AD and FTD may have ventricular expansion (Altmann et al., 2019). The DL-based network may use the boundary of the ventricles, namely, the regions next to them, such as the subcortical regions, corpus callosum and nearby white matter regions, to capture ventricular expansion.

The contribution maps of AD and FTD also showed some differences. The DL-based network gave out a higher weight on the right frontal white matter in FTD, but on the left temporal, bilateral inferior frontal and hippocampal regions in AD. The DL-based network may use the region around the hippocampus to take atrophy information of the hippocampus as well as inferior frontal and temporal regions. It is also worth to note that we found a left-side dominance of the DL-based network contribution in AD, which is consistent with previous studies (Minkova et al., 2017). The white matter and subcortical regions showed very high contribution, and the information may be used to estimate the atrophy of the frontal cortex in FTD, which has been frequently reported in previous studies (Weder et al., 2007). Also, there is also a trend of asymmetry in FTD and this right dominance was especially visualized when compared with AD. This right lateralized pattern is also reported by previous studies (Irwin et al., 2018).

The DL-based network tend to assign higher weight in boundary voxels rather than those within the typical regions, like hippocampus. One possible reason is that the boundary is more important than the inner regions of the atrophied structures to the DL-based network. However, this finding may also indicate that the morphology of nearby white matter regions around the typical atrophied gray matter regions may have potential critical features for AD and FTD, which are ignored previously and needs further investigation.

## Limitation and Conclusion

In conclusion, DL-based classification models eliminate the dependence on professional knowledge and clinical experience and have the ability to solve the enigma of differential diagnosis of diseases. Moreover, they may mine the potential patterns that may be different from human clinicians, which may provide new insight into the understanding of FTD and AD.

There are some limitations that need to be considered. First, the performance on the multiclassification tasks in the looser datasets are not satisfactory. We did not use clinical information to analyze the misclassified samples and improve the model. Second, since the experiment proved that the knowledge learned by the DL network is generalizable, we can extend it to other body parts, diseases and modes. However, considering that the visual interpretation part needs the guidance of clinical experts, we have not carried out that experiments at present. Third, FTD has many subtypes (behavioral variant FTD, semantic variant primary progressive aphasia and non-fluent variant primary progressive aphasia), and generally labeled them as FTD is not conducive to automatic pattern learning. Finally, as FTD and AD are neurodegenerative diseases, the images of these diseases also changed over time, but we did not use the tracking data in the open databases for further study.

### Effect of Spatial and Intensity Normalization

The performance on the multiclassification tasks in the looser datasets were not satisfactory. We supplemented three normalization methods (Table 5) based on scenario 7 to further discuss the effect of spatial and pixel normalization on the resulting: (1). reduced the specified size in spatial normalization; (2). changed the order of spline interpolation in spatial normalization; (3). changed the mean and standard deviation used in pixel normalization.

**TABLE 5:** The effect of spatial and intensity normalization on the classification performance.

Scenario	Spatial normalization		Intensity normalization	Accuracy (%)	Sensitivity (%)		Specificity (%)	
	Specified size	Order			FTD	AD	FTD	AD
7	240 × 256 × 160	0	Individual	66.79	49.29	79.77	58.68	58.35
10	180 × 192 × 120	0	Individual	70.47	56.00	64.47	89.87	83.03
11	120 × 128 × 80	0	Individual	63.36	50.80	59.16	89.40	87.00
12	240 × 256 × 160	1	Individual	68.75	54.80	67.94	65.89	63.91
13	180 × 192 × 120	0	Averaged	58.95	55.60	92.75	85.34	77.80

*The only difference between scenarios 7, 10, 11, 12, and 13 lines in the normalization methods, whose training data composition and test data composition are identical.*

The accuracy, sensitivity and specificity of scenario 10 were all better than that of scenario 7, but the performance of scenario 11 was worse than that of scenarios 7 and 10. It can be inferred that appropriately reducing the size of the original image eased the learning burden of the network, but too small space size led to the loss of useful information. Comparing the performance of scenarios 7 and 12, it can be seen that changing the order of spline interpolation in spatial normalization didn’t improve the performance significantly and was more computationally intensive and time-consuming. Similarly, sharing the same mean and standard deviation in scenario 13 did not simplify the classification problem.

### Early Stages of Disease

We have information which describes the participant’s change in cognitive status from last visit to current visit in the ADNI database. But No similar tracking data is available in the NIFD database. Diagnosis conversion information and the data dictionary in the ADNI database are located on the LONI Image Data Archive^3^ (IDA). Enter your username and password, go to Download, then Study Data. When you click on Assessments, you will see Diagnostic Summary [ADNI1, GO, 2] (DXSUM_PDXCONV_ADNIALL.csv). When you click on Study Info, you will see Data Dictionary [ADNI1, GO, 2] (DATADIC.csv).

MCI is considered a prodromal phase to dementia especially the AD type. The DXCHANGE item in the data dictionary indicates the patient’s disease progression, where DXCHANGE = 4 indicates the change from a normal control to MCI. Filtering all ADNI patients based on this keyword yielded a total of 91 eligible. However, some patients did not have MRI examination before and after the disease transformation. Therefore, the filtered data was not enough to study the early stage classification problem of the same patient.

Nevertheless, we downloaded a standard data collection (adni1: complete 2yr 3T) from ADNI for further analysis. The training set and test set were divided in the same way as other scenarios, please refer to section “Data Collection” for details. The DL network tended to converge after training with about 5,800 images (20 epochs). The accuracy, sensitivity, and specificity of the test set were 58.21, 33.33, and 92.87%, respectively. Specifically, there were 28 NC cases in the test set, and only 2 cases were incorrectly classified as MCI. However, 26 of the 39 MCIs in the test set were missed as NC, which was a very acute failure. MCI is difficult to diagnose due to its rather mild, perhaps using multi-modal data, combining structural data with functional data, and improving the sample size can help solve the problem.

## Data Availability Statement

The original contributions presented in the study are included in the article/supplementary material, further inquiries can be directed to the corresponding author/s.

## Ethics Statement

Ethical review and approval was not required for the study on human participants in accordance with the local legislation and institutional requirements. Written informed consent for participation was not required for this study in accordance with the national legislation and the institutional requirements.

## Author Contributions

JH, ZQ, BZ, XZ, and KH contributed to model design and manuscript preparation. JH, RL, MW, and PL contributed to coding, training and testing the model, and calculating the contribution graph. YW and YG contributed to dataset collection and language proofreading. All authors contributed to the article and approved the submitted version.

## Conflict of Interest

The authors declare that the research was conducted in the absence of any commercial or financial relationships that could be construed as a potential conflict of interest.

## Footnotes

1 https://github.com/BigBug-NJU/FTD_AD_transfer

2 http://adni.loni.usc.edu/methods/documents/

3 https://ida.loni.usc.edu/login.jsp?project=ADNI

## Abbreviations

AD: Alzheimer’s disease
ADNI: Alzheimer’s Disease Neuroimaging Initiative
ADRDA: Alzheimer’s Disease and Related Disorders Association
AD_NC: normal control of ADNI database
bvFTD: behavioral variant FTD
CDR: clinical dementia rating
CNNs: convolutional neural networks
DL: deep learning
FSPGR: fast spoiled gradient-recalled echo
FTD: frontotemporal dementia
FTLDNI: Frontotemporal Lobar Degeneration Neuroimaging Initiative
FTD_NC: normal control of NIFD database
GM: gray matter
ICA: independent component analysis
LDA: linear discriminant analysis
MCI: mild cognitive impairment
ML: machine learning
MMSE: mini-mental state examination
MNI: Montreal Neurological Institute
MPRAGE: magnetization-prepared rapid acquisition with gradient echo
MRI: magnetic resonance imaging
NC: normal control
NIFD: nickname of FTLDNI
NINCDS: National Institute of Neurological and Communicative Disorders and Stroke
PCA: principal component analysis
PET: positron emission tomography
ROC: receiver operating characteristic
ROI: region of interest
SPGR: spoiled gradient-recalled echo
SVM: support vector machine
WM: white matter.
